# Argatroban as an Add-On to rtPA in Acute Ischemic Stroke: A Systematic Review and Meta-Analysis

**DOI:** 10.3390/jcm13020563

**Published:** 2024-01-18

**Authors:** David-Dimitris Chlorogiannis, Theodoros Mavridis, Anastasia Adamou, Ioannis Kyriakoulis, Iliana Stamatiou, Polyxeni Botou, Hui-Sheng Chen, George Ntaios

**Affiliations:** 1 Department of Radiology, Brigham and Women’s Hospital, Harvard Medical School, Boston, MA 02115, USA; 2 1st Department of Neurology, Eginition Hospital, Medical School, National and Kapodistrian University of Athens, 11528 Athens, Greece; mavridismdr@gmail.com; 3 Department of Neurology, Tallaght University Hospital, D24 NR0A Dublin, Ireland; 4 Department of Internal Medicine, School of Health Sciences, University of Thessaly, 41334 Larissa, Greece; anaadamou97@gmail.com (A.A.); ioannis.kyriakoulis@gmail.com (I.K.); 5 Department of Internal Medicine, University Hospital of Alexandroupolis, 68100 Alexandroupolis, Greece; ilianastm@icloud.com; 6 Department of Anaesthesiology, Hippocration General Hospital of Athens, 11527 Athens, Greece; pollybotoump2@gmail.com; 7 Department of Neurology, General Hospital of Northern Theatre Command, Shenyang 110017, China; chszh@aliyun.com

**Keywords:** acute ischemic stroke, intravenous thrombolysis, tPA, endovascular thrombectomy, EVT, direct thrombin inhibitors, DTIs, argatroban, dabigatran, desirudin, lepirudin, bivalirudin, ximelagatran, melagatran

## Abstract

Current treatment options for acute ischemic stroke, including intravenous thrombolysis (IVT) and mechanical thrombectomy, have undoubtedly revolutionized stroke care. The need for additional treatment options has brought into the light direct thrombin inhibitors (DTIs) and, specifically, argatroban as a promising candidate. However, there is uncertainty regarding the safety of adding argatroban to IVT, mainly due to the increased hemorrhagic risk. In this study, we performed a systematic review and meta-analysis examining the safety and efficacy of argatroban as an add-on treatment for IVT. The following databases were searched from inception until the 14th of May 2023: Pubmed/MEDLINE, ClinicalTrials.gov, the EU Clinical Trials Register, EMBASE/Scopus, and the Cochrane Library. Only randomized clinical trials (RCTs) enrolling patients with acute ischemic stroke who underwent IVT evaluating the add-on use of any DTIs were selected for the systematic review and further meta-analysis. The PRISMA guidelines were followed at all stages. Four studies with argatroban were included in the final analysis. Analysis of risk ratio and relative risk shows that the add-on therapy with argatroban seems to be effective and favors a good clinical outcome (mRS 0–2) at 90 days, similar to that of alteplase. All studies showed a low pooled incidence of symptomatic intracerebral hemorrhage (5%), parenchymal hematoma (3%), and other major bleeding (1%). Argatroban as an add-on treatment to IVT seems not to be associated with excessive bleeding risk; however, its efficacy remains unproven. According to this synopsis of the currently available evidence, it is premature to use argatroban as an add-on to IVT treatment outside the current clinical trial setting.

## 1. Introduction

Acute ischemic stroke (AIS) constitutes the most common type of stroke, bearing high morbidity, mortality, and disability [1]. While intravenous thrombolysis remains the cornerstone of the treatment of AIS, only a third of the patients treated with IVT will benefit from treatment, while many patients (14–34%) will deteriorate after initial recanalization and remain disabled due to arterial reocclusion [2,3,4]. Furthermore, there is evidence that rt-PA has minimal to no effect in large thrombi and in larger, more proximal occlusions, thus failing to achieve reperfusion [5]. Moreover, even with the addition of endovascular thrombectomy, roughly half of the patients achieved functional independence of modified Rankin scale (mRS) 0–2 at 90 days [6].

Therefore, there is an additional clinical need for new and effective treatments that will improve reperfusion and reduce reocclusion rates, thus leading to better outcomes. Direct thrombin inhibitors (DTIs), like argatroban, have been previously used in the treatment of AIS, both atherosclerotic and cardioembolic, specifically in the Asian population. The choice of argatroban over other DTIs lies in its pharmacokinetic properties and patient surveillance after administration. Following initiation through the intravenous route, argatroban achieves a state of equilibrium in its plasma levels within a time frame of 1–3 h and induces its anticoagulant effect [7,8]. It manifests a linear dose-dependent response in assays such as the activated partial thromboplastin time (aPTT), affording a wide safety margin [9]. Upon cessation of argatroban, within 2–4 h, the aPTT promptly returns to its baseline values. Moreover, argatroban has been proposed as an add-on to thrombolytic therapy for the treatment of AIS due to evidence of promising results in pre-clinical studies [10]. These results were also translated into early-phase and multicenter trials which examined the safety and benefit of argatroban as an add-on therapy to thrombolysis and endovascular therapy [11,12]. However, a recent randomized controlled trial did not replicate these favorable results [13]. Furthermore, there is uncertainty regarding the safety of adding DTIs to IVT, mainly due to the increased hemorrhagic risk. 

Herein, we aim to encapsulate the current available funds of knowledge concerning the role of argatroban as an additional treatment to thrombolytic therapy for the treatment of acute ischemic stroke and examine its safety and role in improving neurological outcomes.

## 2. Methods

The design of this systematic review followed the PRISMA (Preferred Reporting Items for Systematic Reviews and Meta-Analyses) guidelines. The protocol of the study has been prospectively registered in PROSPERO (CRD42023423630). No ethical approval was required, since no new patients were involved.

### 2.1. Eligibility Criteria 

To be considered for inclusion in the systematic review, a study had to meet the following inclusion criteria: (i) a cohort of adult patients presenting with acute ischemic stroke who received an intravenous recombinant tissue-type plasminogen activator; (ii) examine either the safety or efficacy of the addition of a direct thrombin inhibitor; (iii) and/or compare it with standard-of-care therapy. Outcomes of interest included functional outcome at 90 days, symptomatic intracranial hemorrhage, parenchymal hematoma type 2, and major systemic bleeding. Cohort studies (both prospective and retrospective) and randomized controlled trials (RCTs) were deemed eligible. Finally, we excluded case reports, case series, case–control, cross-sectional, descriptive, animal, and in vitro studies.

### 2.2. Information Sources and Search Strategy

The literature search was conducted by systematically searching Medline (via PubMed), EMBASE (via Scopus), CENTRAL (the Cochrane Central Register of Controlled Trials), the Clinicals.gov Trials registry, and Clinicaltrialsregister.eu; in addition, the full reference lists of the retrieved studies were also searched to identify additional articles (the “snowball” method). Databases were searched from inception. A combination of keywords and MeSH (Medical Subject Headings) terms was used to define the search strategies. The detailed applied algorithms are available in Supplementary Table S1. The date of the last search was set at 14 May 2023, without applying language restriction. 

### 2.3. Selection Process

The study selection process comprised a sequential three-step approach. Initially, the titles and abstracts of all electronic records were evaluated to identify potentially eligible studies. Subsequently, articles deemed possibly eligible were obtained in full-text form. Thereafter, studies failing to report outcomes of interest or meeting any of the exclusion criteria were excluded. The selection of studies was independently conducted by two researchers, with any disagreements resolved by a third independent researcher.

### 2.4. Data Extraction and Data Items

Pre-piloted forms were used for extraction of the following data from the included studies: year of publication, country, study period, design, sample size, eligibility criteria, participants’ median age and sex, duration of follow-up, NIHSS score, ASPECT score at diagnosis, and direct thrombin inhibitor. Information regarding the outcomes of interest (functional outcome at 90 days, parenchymal hematoma type 2, and major systemic bleeding) was also collected. Two independent authors collected the data, resolving any potential discrepancies after consulting a third author.

### 2.5. Risk of Bias Assessment

The risk of bias of the included RCTs was evaluated using the RoB-2 tool [14], assessing the following domains: randomization, deviations from intended interventions, missing outcome data, measurement of outcomes, and selection of the reported results. The risk of bias was judged as low, moderate, serious, or critical. The process of quality assessment was conducted independently by two researchers, blinded to each other, and any discrepancy was resolved through the consensus of all authors. 

### 2.6. Statistical Analysis

Data analysis was performed in R- 2023.03.1 + 446 (R foundation) (package “*meta*”) version [15,16,17,18]. Statistical significance was defined by a two-sided *p*-value threshold of 0.05. Continuous variables were summarized using means and standard deviations, while categorical variables were summarized by using absolute values and relative frequencies. The Wan et al. method was used to estimate the means and SDs of continuous variables whenever medians and ranges and medians and interquartile ranges were provided, respectively [19]. Risk ratios with 95% confidence intervals (CIs) were used as a measure of effect. A random-effect model (the Mantel–Haenzel procedure) was used to estimate the pooled ORs for RCTs and observational trials independently and for mixed analysis [20]. Inverse variance weights were used in all cases. Inconsistency test (I^2^) statistics were used to assess the heterogeneity (I^2^ = 100% × (Q − df)/Q, where Q = χ^2^ (Cochran’s heterogeneity statistic) and df = degrees of freedom), where I^2^ ≤ 25% signifies low heterogeneity, I^2^ ≤ 50% is moderate heterogeneity, and I^2^ > 50% is considered high heterogeneity [21]. 

### 2.7. Certainty of Evidence

The GRADE approach was used to evaluate the certainty of evidence per outcome. Specifically, the certainty of evidence was judged as very low, low, moderate, or high by taking into account the following domains: study limitations, inconsistency, indirectness, imprecision, and publication bias.

## 3. Results

### 3.1. Study Selection

**Figure 1:**
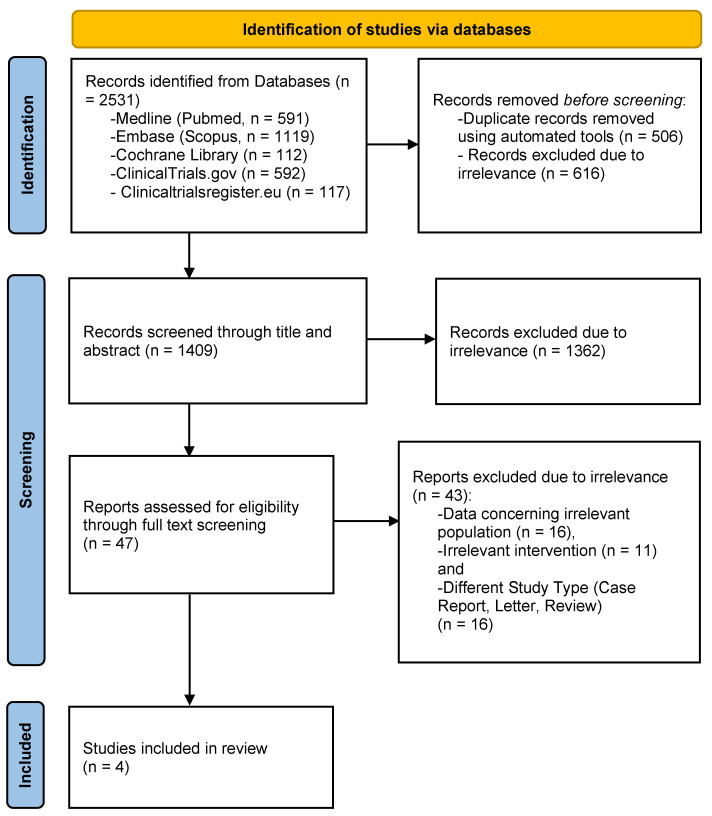
PRISMA flowchart for study selection.

The process of study selection is schematically depicted in a PRISMA flowchart (Figure 1). The literature search resulted in a total of 2531 records. After the removal of duplicates, 1409 articles were screened for eligibility and 47 of them were retrieved in full-text form. Subsequently, 43 studies were excluded because they reported data concerning different populations (n = 16) or interventions (n = 11), or were of different research study types (review, case report, or letter to the editor; n = 16). As a result, a total of four studies were included in the meta-analysis, comprising 915 participants [11,12,13,22]. Of note, even though the original search included every relevant article concerning the addition of DTIs to alteplase, the four studies only report the results of argatroban. The total number of patients who received argatroban with concomitant administration of alteplase was 519, while 396 patients received only alteplase.

### 3.2. Included Studies

The methodological characteristics of the included studies are presented in Table 1. Τwo were RCTs [13,22] and two were prospective cohorts [11,12]. Every study tested argatroban as the direct oral anticoagulant adjunct to alteplase. The argatroban infusion protocol was as follows: Argatroban IV bolus 100 mcg/kg, followed by Argatroban IV Infusion 1 mcg/kg/min (low dose) for 48 h or 3 mcg/kg/min (high dose). No RCT study was judged to be at critical risk of bias (Supplementary Figure S1). 

### 3.3. Neurological Functional Outcome at 90 Days

**Figure 2:**
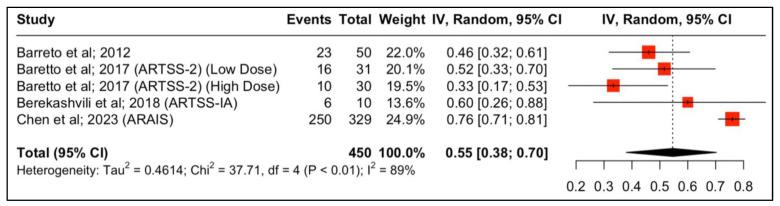
Proportions of patients with improved neurological function (mRS 0–2) at 90 days post-treatment.

**Figure 3:**
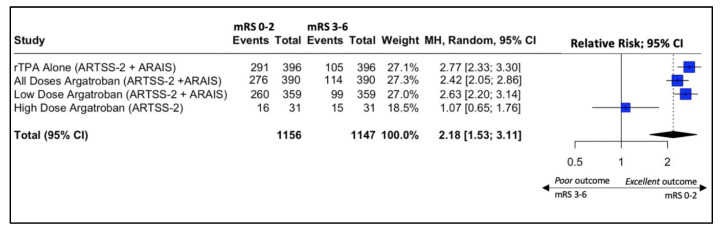
Forest plot of the association of the addition of argatroban to standard treatment (rtPA) with clinical outcome at 90 days.

Four studies reported outcomes regarding the neurological functional improvement at 90 days. The results are depicted in Figure 2. The study by Baretto et al. (ARTSS-2) was split into two populations due to being a multiarm study evaluating different doses of argatroban after the initial bolus infusion, either 1 (low dose) or 3 mcg/kg per minute (high dose), for 48 h. In this multicenter study, 6 patients (21%) in the rtPA alone group, 10 (32%) in the low-dose argatroban plus rtPA group, and 9 (30%) in the high-dose argatroban plus rtPA group had an mRS of 0–1 at 90-day follow-up. The addition of argatroban indicated a 79% probability to increase the percent of patients with a score of 0–1 on the modified Rankin scale at 90 days compared to rtPA alone (risk ratio (RR), 95% CI: 1.34 (0.68, 2.76), 79%, *p*-value > 0.05) [22]. Moreover, with regard to clinical independence, 11 (37.9%), 10 (33.3%), and 16 (51.6%) patients achieved an mRS of 0–2 at 90 days in the rtPA alone low-dose and high-dose argatroban + alteplase groups, respectively (Figure 3).

The ARTSS-IA trial, a single-arm feasibility study, examined the addition of argatroban in patients with intracranial large vessel occlusions who received standard-dose rtPA and underwent endovascular thrombectomy in the first 6 h of stroke onset. In this safety study, 6 out of the 10 patients (60%) had a modified Rankin scale of 0–2 at 90 days.

**Figure 4:**
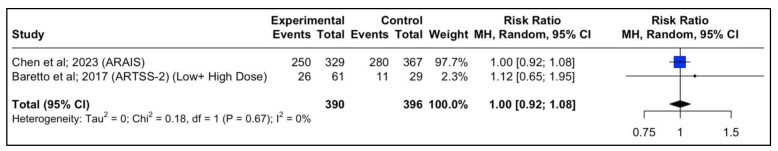
Forest plot of the pooled estimated risk ratio of Argatroban add-on to rtPA vs. rtPA alone in patients with acute ischemic stroke and good neurological outcome (mRS 0–2) at 90 days.

In the ARAIS trial, a larger, multicenter, randomized trial, 250 of 329 participants (76%) in the argatroban plus alteplase group vs. 280 of 367 (76.3%) in the alteplase alone group had a modified Rankin scale of 0–2 at 90 days (risk difference, −0.3% (95% CI: −6.6% to 6.0%, *p*-value > 0.05; RR, 0.98 (95% CI, 0.88 to 1.10); *p*-value > 0.05). When the two results from the RCTs were pooled together, the result was similar between the two groups (RR, 1.00 (95% CI, 0.92 to 1.09); *p*-value > 0.05) (Figure 4). 

Forest plot analysis of risk ratio and relative risk (Figure 3 and Figure 4) shows that the add-on therapy to alteplase with argatroban is effective and favors a good clinical outcome (mRS 0–2) at 90 days, similar to that of alteplase. Only the high dose of argatroban + alteplase did not achieve statistical significance regarding the functional outcome. That may also be due to the higher baseline NIHSS and the small number of patients.

The quality of the evidence is evaluated as moderate due to variability in study designs (only two RCTs with conflicting results), inconsistency, and possible publication bias (Supplementary Figure S2).

### 3.4. Parenchymal Hematoma Type-2, Intracranial Hemorrhage, and Major Systemic Bleeding Rates

**Figure 5:**
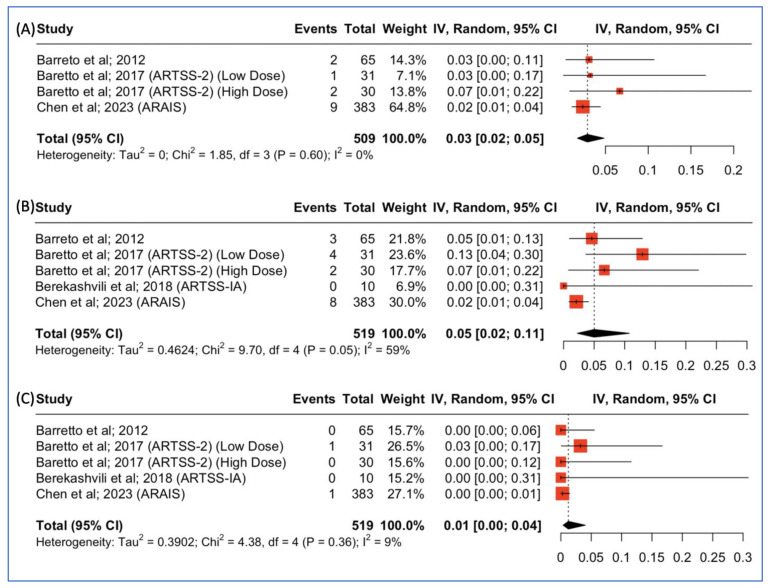
(**A**) Proportions of patients with parenchymal hematoma type 2 post-treatment. (**B**) Proportions of patients with intracranial hemorrhage post-treatment. (**C**) Proportions of patients with major systemic bleeding post-treatment.

Three studies reported outcomes concerning the incidence of parenchymal hematoma type 2 post-combined argatroban and tPA administration for patients with acute ischemic stroke. The pooled incidence was 14/509 (3%, 95% CI: 2–5%), as depicted in Figure 5A. Moreover, all four studies reported outcomes concerning the incidence of intracranial hemorrhage post-combined argatroban and tPA administration for patients with acute ischemic stroke. The pooled incidence was 17/519 (5%, 95% CI: 2–11%), as depicted in Figure 5B. Lastly, all four studies reported outcomes concerning the incidence of major systemic bleeding post-combined argatroban and tPA administration for patients with acute ischemic stroke. The pooled incidence was 2/519 (1%, 95% CI: 0–4%), as depicted in Figure 5C.

## 4. Discussion

This systematic review and proportional analysis showed that the addition of argatroban in IV rtPA treatment for acute ischemic stroke (AIS) is relatively safe regarding symptomatic parenchymal hematoma type 2, intracranial hemorrhage, and major systemic bleeding. However, it did not improve outcomes compared to IVT alone.

Argatroban is a small synthetic L-arginine derivative (molecular weight = 527 D) that was introduced in 1970 [23,24,25,26] and acts as a direct thrombin inhibitor through a reversible interaction with the catalytic site of thrombin [9,27,28]. Regarding its potential use in ischemic stroke treatment, the first preliminary positive evidence resulted from basic research studies of middle cerebral artery occlusion models [29,30,31]. These studies underlined the beneficial effects of argatroban in protecting the neurovascular unit and suppressing the extension of the infraction within the penumbra. This effect was exerted by maintaining the blood vessels patent and inhibiting microthrombogenesis.

Additionally, thrombin inhibition plays a vital role in safeguarding the vascular endothelium from injury, thereby promoting the production of endogenous plasminogen activators [32]. In clinical practice, argatroban has been widely used as a secondary treatment of AIS, both atherosclerotic and cardioembolic, particularly in Asian countries, such as China and Japan [33,34,35]. The main hypothesis was that the addition of argatroban on top of rt-PA would have the potential to ameliorate the “no-reflow” phenomenon within the microcirculation. This combined approach may lead to an acceleration and thoroughness of recanalization, while also reducing the risk of reocclusion, ultimately resulting in a decrease in the extent of infarction. The first encouraging results came from preclinical studies, where argatroban plus rtPA enhanced the thrombolytic effect, prolonged the arterial recanalization, and prevented reocclusion without increasing the hemorrhagic transformation [10,36].

Even though those studies were heterogenous and had conflicting results regarding effectiveness, they exhibited similar safety results, which is also replicated in our proportional analysis [11,12,13,22]. The different study designs with respect to the population, the severity of the NIHSS, the dosage of argatroban, the duration of infusion, the definitions of sICH, and the primary and secondary endpoints are some of the reasons that can explain the difference in their results. The first multicenter, single-arm pilot study was performed to assess the safety profile of administering argatroban in addition to the standard dose of rt-PA for ischemic stroke. This study showed a low incidence of symptomatic intracranial hemorrhage (sICH) in patients [12] (Table 1, Figure 5). Moreover, 40% of the patients achieved complete recanalization within two hours, a significantly higher rate compared to historical controls treated with rt-PA alone (18%). Additionally, almost half of the patients demonstrated a good clinical outcome (mRS 0–2) (Table 1, Figure 2) [12].

The second study (ARTSS-2) was a three-arm, multicenter, randomized, exploratory trial that evaluated the safety and the probability of a favorable outcome with the addition of argatroban (administered in two different dose regimens) to rtPA in patients with AIS. The percentage of patients achieving a good clinical outcome (mRS 0–2), in the rt-PA alone, low-dose argatroban, and high-dose argatroban groups, was comparable to the previous study (Table 1, Figure 2). With regard to sICH, both ARTSS-2 and the previous study [22] used the more conservative sICH definition of NINDS [37]. Taking into consideration the SITS-MOST definition of sICH [38], the incidence of sICH was even lower (2.4%) in patients who received either dosage regime of argatroban plus alteplase and comparable with that of rtPA alone.

The subsequent ARTSS-IA study was a single-arm feasibility and safety study and included patients with intracranial large vessel occlusions who received standard-dose r-tPA and underwent EVT within 6 h of stroke onset. This study used a different protocol combining argatroban with IVT and EVT in patients with AIS and large vessel occlusion [11].

The duration of infusion was less than in the previous studies (12 vs. 48 h) and the researchers used the newer SITS-MOST definition for sICH (vs. NINDS in the previous studies). A high percentage (>50%) of the patients achieved functional independence (mRS 0–2), with no reported deaths or sICH (Table 1). However, two patients did exhibit asymptomatic intracranial hemorrhage. The same results were also found in a post hoc analysis of the Multi-MERCI trial for stroke endarterectomy. In that study, a comparison was made between cases that received intravenous heparin (n = 24; median dose, 3000 units) and those that did not (n = 27). The analysis revealed no increased risk of intracranial hemorrhage (ICH) (risk difference, −6.94% (95% CI: −33.9% to −20.01%, *p*-value = 0.7766)) or death (risk difference, −8.97% (95% CI: −35.74% to −17.79%, *p*-value = 0.57)) associated with the use of heparin [39]. On the contrary, procedural heparin was found to be independently linked with favorable neurological clinical outcomes at the 90-day mark, a result that was clinically significant. The aforementioned results were in agreement with those from the ARTSS-IA study, and that could be explained by considering the similarities between unfractionated heparin and argatroban [11].

The most recent and largest study, Argatroban Plus Recombinant Tissue-Type Plasminogen Activator for AIS (ARAIS), was a multicenter, open-label, randomized trial that evaluated the efficacy of argatroban plus rtPA in patients with AIS within 4.5 h from symptom onset [13]. The protocol of ARAIS included low-dose argatroban with IVT versus IVT alone in patients with AIS. This larger study failed to show any difference in clinical improvement between the two groups regarding excellent (mRS 0–1) and good (mRS 0–2) clinical outcomes at 90-days, as is depicted in Table 1 and Figure 2.

The incidence of adverse events (including bleeding events) was comparable between the two groups. Notably, the rates of bleeding events were similar in both the argatroban plus alteplase group and the alteplase alone group (2.1% vs. 1.8%, respectively), which aligns with findings from prior historical studies [11,12,22]. There was a difference in the percentage of patients receiving both argatroban and alteplase that reached an independency score of mRS 0–2 in the three studies [12,13,22], as seen in Table 1. Those differences can be explained, as Chen et al. pointed out, by the difference in numbers and NIHSS scores, since the first two studies had a higher median NIHSS compared to the ARAIS trial. More specifically, a substantial disparity was evident in the proportion of patients achieving an excellent functional outcome when comparing the anticipated values utilized in the sample size calculation (21%) and previous historical data (31%) with the observed values in this trial (64%). This variation could potentially be ascribed to the composition of the enrolled population, characterized by individuals presenting with milder neurologic deficits, as reflected in a lower median NIHSS than the one observed in previous studies (Table 1).

Furthermore, LVO patients were not a mandatory inclusion criterion for the ARAIS trial, which is in accordance with the difference in NIHSS scores, thus resulting also in a higher proportion of patients who benefited with or without the addition of argatroban. Of note is that even the percentage of the control group (patients receiving alteplase with mRS 0–2) was higher compared to historical data regarding the efficacy of alteplase in AIS (76.3% vs. 38.6% achieved ambulation independence, i.e., mRS 0–2) [40]. That difference can also be seen when setting side by side the number of patients who achieved an excellent clinical outcome of mRS 0–1 in the ARAIS trials and a meta-analysis of nine trials (64.9% vs. 31%, respectively) [41]. All of the aforementioned factors point to a hypothesis that argatroban plus alteplase may increase recanalization and decrease the reocclusion rates of large artery occlusion, resulting in better neurologic improvement [12,22], though further studies are needed. Moreover, in the setting of LVO, the addition of argatroban on top of alteplase and EVT resulted in an independency (mRS 0–2) at 90 days of 60%, which is higher than that reported in the OPUS-REACH registry (43% IVT plus EVT) [11,42].

Even though it requires good coordination of the stroke team, concomitant argatroban seems to be feasible in certain settings, does not delay EVT provision, and produces high rates of recanalization [11]. Nevertheless, more trials are necessary to investigate the potential impact of administering alteplase in combination with argatroban in patients with large artery occlusion. In particular, such studies could assess the efficacy and safety of argatroban as an add-on to alteplase for acute ischemic stroke (AIS) cases with LVO where other factors (e.g., lack of transportation and no EVT capabilities) preclude thrombectomy (drip and ship) as a treatment option. Despite the discrepancies found between those studies regarding the efficacy of adjunctive therapy with argatroban, this study underlines an agreement about its safety. All studies, even though different definitions of sICH [37,38] were used, showed a low incidence of sICH, PH2, and other major bleeding. That incidence is comparable to the 3.7% sICH rate reported in a recent meta-analysis of 3391 rt-PA-treated strokes from nine randomized trials and lower than the rate of NINDS-tPA (6.4%), which played a pivotal role in the FDA approval of r-tPA [37]. Based on this similarity regarding bleeding events, we postulate that the concomitant administration of Argatroban with r-tPA could possibly be safe. Furthermore, even the higher-dose arm of Argatroban showed promising results, with no increase in bleeding events and improved clinical outcomes. The Multi-arm Optimization of Stroke Thrombolysis (MOST; *NCT03735979*) study is currently ongoing, and its outcomes are expected to elucidate the efficacy and safety of argatroban plus alteplase therapy [43].

This systematic review and meta-analysis is not without limitations. Firstly, the quantitative synthesis was performed using study-level data and not with patient-level data, which reveals that no adjustment for baseline demographic factors was made, which could have introduced possible confounding bias, with high heterogeneity between them (Figure 2). However, no significant heterogeneity between the RCT studies was noted (Figure 3). Secondly, this study included a limited number of clinical trials, with some being relatively small and lacking control groups. However, the overall outcomes of this study mirror a representation of real-world data and represent the existing funds of knowledge. Nevertheless, this systematic review provides a holistic and comprehensive overview of the existing funds of knowledge while tackling the gray areas to guide clinicians for potential future research.

## 5. Conclusions

Argatroban as an add-on treatment to IVT seems to not be associated with excessive bleeding risk; however, its efficacy remains unproven. According to this synopsis of the currently available evidence, it is premature to use argatroban as an add-on to IVT treatment outside the current clinical trial setting.

## Figures and Tables

**Table 1:** Studies and patients’ characteristics.

Author		Barretto et al. [12]	Barretto et al. [22]		Berekashvili et al. [11]	Chen et al. [13]
**Year**		2012	2017		2018	2023
**Type of study**		Prospective observational	Randomized control trial		Prospective observational	Randomized control trial
**Study period**		May 2003–August 2010	December 2011–March 2015		June 2015–May 2016	January 2019–October 2021
**Country**		Multinational	USA, UK		USA	China
**Number of patients (n)**		65	90		32	817
**Argatroban + thrombolysis (n (%))**		65 (100)	61 (67.8)		10 (31.3)	402 (49.2)
**Thrombolysis (n (%))**		0 (0)	29 (32.2)		22 (68.7)	415 (50.8)
**Duration of follow-up (days)**		7	90		90	90
**Female gender (n (%))**	*Argatroban + thrombolysis*	36 (55.4)	28 (31.1)		NA	126 (31.3)
	*Thrombolysis*	0 (0)	12 (41.4)		NA	112 (27)
**Age (years (mean + SD))**	*Argatroban + thrombolysis*	63 ± 14	69 ± 14		69 ± 12.6	66 ± 10
	*Thrombolysis*	NA	69 ± 15		NA	64 ± 10
**NIHSS**	*Argatroban + thrombolysis*	13 (3–22)	14		19.5 (12–24)	9 (7–12)
	*Thrombolysis*	NA	15		NA	9 (6–12)
**ASPECTS**	*Argatroban + thrombolysis*	10 (4–10)	9 (8–10)		9(8–9)	9 (8–10)
	*Thrombolysis*	NA	10 (8–10)		NA	9 (8–10)
**Time from event to IVT (min (mean + SD))**	*Argatroban + thrombolysis*	130.7 ± 57.6	134 ± 52		45.7 ± 12	162.7 ± 65.5
	*Thrombolysis*	NA	114 ± 43		NA	157.3 ± 64
**sICH (%)**	*Argatroban + thrombolysis*	4.6	13 *	7 **	0	2.1
**mRS 0–2 (%)**	*Argatroban + thrombolysis*	46	33.3 *	51.6 **	60	76

**Abbreviations:** USA: United States of America, UK: United Kingdom, NA: not available, NIHSS: National Institutes of Health Stroke Scale, ASPECTS: Alberta Stroke Program Early CT Score, IVT: intravenous thrombolysis, *: low-dose argatroban, **: high-dose argatroban. Results are presented as medians (IQRs), unless otherwise indicated.

## Data Availability

No new data were created or analyzed in this study. Data sharing is not applicable to this article.

## Supplementary Materials

The following supporting information can be downloaded at: https://www.mdpi.com/article/10.3390/jcm13020563/s1, Table S1: Detailed Search Strategy in the Major Databases.; Figure S1: RoB-1 Quality assessment of the RCT studies depicting low risk of overall bias for both studies.; Figure S2: GRADE Summary of Findings Table.
