# Supplementary material for: Argatroban as an Add-On to rtPA in Acute Ischemic Stroke: A Systematic Review and Meta-Analysis
Source: J Clin Med. 2024 Jan 18;13(2):563. doi: 10.3390/jcm13020563 (PMC10816854; doi:10.3390/jcm13020563)
Supplement: Supplementary file 1 [file jcm-13-00563-s001.zip › jcm-2771575-supplementary.pdf]

Supplementary Material: Argatroban as an add-on to rTPA in acute ischemic stroke: A Systematic Review and Meta-Analysis.

Supplementary Table S1. Detailed Search Strategy in the Major Databases

Database Screened and Results	Search Terms	Limits Used
**MEDLINE**<br>(591 Results)	*((((((((((((((((((((((tpa) OR (thrombolysis)) OR (intravenous thrombolysis)) OR (rtpa)) OR (alteplase)) OR (tenecteplase)) OR (Streptokinase)) OR (Reteplase)) OR (Urokinase)) OR (Prourokinase)) OR (Anistreplase)) OR (TNK-tPA)) OR (Anisoylated purified streptokinase activator complex)) OR (APSAC)) OR (pro-urokinase)) OR (scu-PA)) OR (r-PA)) OR (tissue plasminogen activator)) OR (recombinant tissue plasminogen activator)) OR (single-chain urokinase plasminogen activator)) OR (Staphylokinase)) OR (Sak)) AND (((((((stroke) OR (ischemic stroke)) OR (ischaemic stroke)) OR (acute ischemic stroke)) OR (acute stroke)) OR (acute ischaemic stroke)) OR (cerebrovascular accident*))) AND (((((((((((dti) OR (direct thrombin inhibitor*)) OR (Dabigatran)) OR (Argatroban)) OR (Melagatran)) OR (Ximelagatran)) OR (Bivalirudin)) OR (Lepirudin)) OR (Desirudin)) OR (antithrombin*)) OR (thrombin inhibitor*))*	No Limits
**EMBASE (*Scopus*)**<br>(1119 Results)	*TITLE-ABS-KEY (tpa) OR (thrombolysis)) OR ("intravenous thrombolysis")) OR (rtpa)) OR (alteplase)) OR (tenecteplase)) OR (streptokinase)) OR (reteplase)) OR (urokinase)) OR (prourokinase)) OR (anistreplase)) OR (tnk-tpa)) OR ("Anisoylated purified streptokinase activator complex")) OR (apsac)) OR (pro-urokinase)) OR (scu-pa)) OR (r-pa)) OR ("tissue plasminogen activator")) OR ("recombinant tissue plasminogen activator")) OR ("single-chain urokinase plasminogen activator")) OR (staphylokinase)) OR (sak)) AND (((((((stroke) OR ("ischemic stroke")) OR ("ischaemic stroke")) OR ("acute ischemic stroke")) OR ("acute*	No Limits

	stroke")) OR ("acute ischaemic stroke")) OR ("cerebrovascular accident"))) AND (((((((((((dti) OR ("direct thrombin inhibitor*")) OR (dabigatran)) OR (argatroban)) OR (melagatran)) OR (ximelagatran)) OR (bivalirudin)) OR (lepirudin)) OR (desirudin)) OR (antithrombin*)) OR ("thrombin inhibitor*")))*	
**Cochrane Library**<br>(112 Results)	*(((((((((((((((((((((((tpa) OR (thrombolysis)) OR ("intravenous thrombolysis")) OR (rtpa)) OR (alteplase)) OR (tenecteplase)) OR (Streptokinase)) OR (Reteplase)) OR (Urokinase)) OR (Prourokinase)) OR (Anistreplase)) OR (TNK-tPA)) OR ("Anisoylated purified streptokinase activator complex")) OR (APSAC)) OR (pro-urokinase)) OR (scu-PA)) OR (r-PA)) OR ("tissue plasminogen activator")) OR ("recombinant tissue plasminogen activator")) OR ("single-chain urokinase plasminogen activator")) OR (Staphylokinase)) OR (Sak)) AND (((((((stroke) OR ("ischemic stroke")) OR ("ischaemic stroke")) OR ("acute ischemic stroke")) OR ("acute stroke")) OR ("acute ischaemic stroke")) OR (("cerebrovascular" NEXT accident*)))) AND (((((((((((dti) OR (("direct thrombin" NEXT inhibitor*))) OR (Dabigatran)) OR (Argatroban)) OR (Melagatran)) OR (Ximelagatran)) OR (Bivalirudin)) OR (Lepirudin)) OR (Desirudin)) OR (antithrombin*)) OR (("thrombin" NEXT inhibitor*)))*	No Limits
**Clinicaltrials.gov**<br>(592 Results)	*Desirudin, Lepirudin, Bivalirudin, Ximelagatran, Melagatran, Argatroban, Dabigatran*	No Limits
**Clinicaltrialsregister.eu**<br>(117 Results)	*Desirudin, Lepirudin, Bivalirudin, Ximelagatran, Melagatran, Argatroban, Dabigatran*	No Limits

Supplementary Figure S1. RoB-1 Quality assessment of the RCT studies depicting low risk of overall bias for both studies.

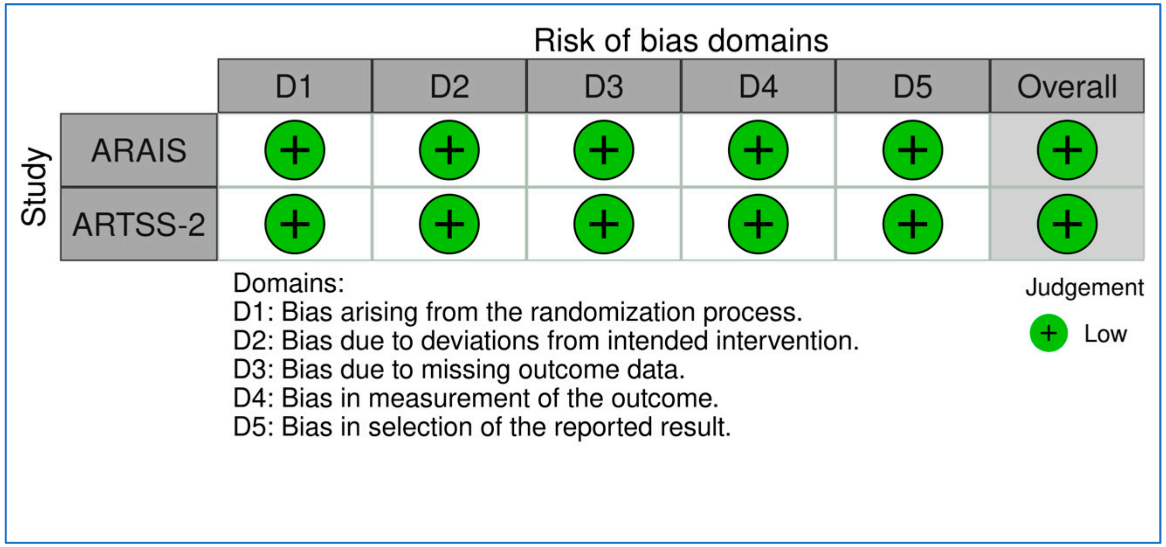

Supplementary Figure S2. GRADE Summary of Findings Table.

Argatroban as an add-on to rtPA in acute ischemic stroke: A Systematic Review and Meta-Analysis
Patients or population: Patients with acute ischemic stroke who received intravenous recombinant tissue-type plasminogen activator.

Settings: Hospital

Intervention: Argatroban

Comparison: No Argatroban

Outcome	N of participants (Studies)	Certainty of Evidence	Relative Effect (95% CI)
Good neurological functional Outcome (mRS 0-2) at 90 days	(2 Randomized Controlled Studies)	MODERATE[1] ⊕⊕⊕◯	Risk Ratio (RR) 1.00 (0.92-1.08)

The corresponding risk (and its 95% confidence interval) is based on the assumed risk in the comparison group and the relative effect of the intervention (and its 95% CI).
CI: confidence interval**; RR:** risk ratio

GRADE Working Group grades of evidence
High quality: Further research is very unlikely to change our confidence in the estimate of effect.
Moderate: quality: Further research is likely to have an important impact on our confidence in the estimate of effect and may change the estimate.
Low quality: Further research is very likely to have an important impact on our confidence in the estimate of effect and is likely to change the estimate.
Very low quality: We are very uncertain about the estimate.

EXPLANATION
[1] Only two studies with conflicting results
Point estimates vary widely across studies (Inconsistency) and
Publication Bias
